# Contested borders: organized crime, governance, and bordering practices in Colombia-Venezuela borderlands

**DOI:** 10.1007/s12117-020-09399-3

**Published:** 2020-11-12

**Authors:** Viviana García Pinzón, Jorge Mantilla

**Affiliations:** 1 German Institute for Global and Area Studies, Hamburg. Neuer Jungfernstieg 21, 20354 Hamburg, Germany; 2 Department of Criminology, Law, and Justice, University of Illinois at Chicago, Chicago, IL USA

**Keywords:** Organized crime, Borderlands, Political geography, Space, Colombia, Venezuela

## Abstract

Based on the conceptualizations of organized crime as both an enterprise and a form of governance, borderland as a spatial category, and borders as institutions, this paper looks at the politics of bordering practices by organized crime in the Colombian-Venezuelan borderlands. It posits that contrary to the common assumptions about transnational organized crime, criminal organizations not only blur or erode the border but rather enforce it to their own benefit. In doing so, these groups set norms to regulate socio-spatial practices, informal and illegal economies, and migration flows, creating overlapping social orders and, lastly, (re)shaping the borderland. Theoretically, the analysis brings together insights from political geography, border studies, and organized crime literature, while empirically, it draws on direct observation, criminal justice data, and in-depth interviews.

## Introduction

The Colombian-Venezuelan border is currently considered one of the most dangerous borderlands worldwide as turf wars, among a plethora of non–state armed actors, have spiked violence. Meanwhile, the exodus of Venezuelan nationals fleeing the political and economic crisis in their country has reached similar proportions to those provoked by the civil war in Syria, and it constitutes the largest migration wave in Latin American recent history (ACNUR 2019). More than 4,8 million people left Venezuela over the last decade. Of them, at least 1,8 million live in Colombia, and the majority entered the country through land borders. The massive influx of migrants and the subsequent humanitarian crisis compound the complex security scenario of a borderland historically characterized by poverty and exclusion, state neglect, the thriving of illegal economies, and non–state forms of order and governance (Idler 2019). Further complicating matters, rifts between the governments of both countries have impeded the development of concerted efforts to address the situation in the borderlands.

Against the backdrop of the strained bi-lateral relation, actions such as the closure of borders and blockades to the transnational flow of goods and people are part of the repertoire of tools employed by national governments. That is how, in a decision that remains in force, the Bolivarian government of Venezuela decided to close the border in an alleged national security maneuver against Colombian paramilitaries and smuggling gangs in 2015.^1^ Border enforcement by the Venezuelan state had a marginal impact on deterring irregular immigration and crime dynamics. On the contrary, it contributed to further channeling the flows of people and goods through illegal paths controlled by non–state armed groups. These groups, in turn, seized the opportunity to strengthen their power to regulate cross-border activities and to hold control over the migrant population; all this with direct and profound effects on socio-spatial practices and the (re)production of the borderland.

The engagement of organized crime in bordering practices and the complexities of its relationship with the spatial particularities of borderlands are aspects that remain underexplored. On the one hand, borderlands have been marginal in the literature on organized crime. Canonical perspectives posit that organized crime groups —henceforth OCGs— erode borders, menace the state, and put territorial sovereignty at stake and should be therefore considered a global threat to the contemporary world order (Payan 2006; Salehyan 2011). In contrast, other research has shown that OCGs and other non–state armed actors, such as smuggling networks, can play a key role in state-building process and governance capacities across borders (Goodhand 2005; Andreas 2013; Shortland and Varese 2016), or even be considered service providers worth of trust by those who make a living out of illegal markets (Sanchez 2015; Tinti and Reitano 2017). Despite the developments in the analysis of organized crime in the contexts of borderlands, this strand of scholarship lacks the tools to theorize on space and border analysis.

On the other hand, political geography and border studies have come to a great length in developing concepts to grasp the complexity and manifold aspects of border and borderlands across time and space. In this regard, actions related to the creation, management, and transformation of borders, this is ‘bordering practices’ (Parker and Adler-Nissen 2012), have been at the core of this literature. It recognizes that bordering practices are not a realm exclusive to the state but that other actors —ranging from local communities to private companies, and NGOs (Cooper et al. 2016; Gavrilis 2008; Lamb 2014)— are involved in the co-production of borders. However, this strand of scholarship does not acknowledge the capacity of OCGs to also engage in bordering practices and the configuration of borderlands. It regards OCGs as actors that either seek to circumvent the border to carry out their activities or that get involved in cross–border activity to get material gains and for strategic purposes, what Rumford (2014) calls “opportunistic use of borders.” While we agree with the idea that the relationship between organized crime and borders its etched by economic and strategic motivations, we contend that organized crime has deeper consequences on borders and borderlands than recognized in this strand of literature. In fact, we consider that organized crime constitutes an avenue to explore the social and spatial (re)production of borders.

Based on these assumptions, the present paper brings insights from political geography and border studies into dialogue with the literature on organized crime and non–state governance to analyze the role of organized crime in bordering practices in the case of the Colombia-Venezuelan borderlands. We argue that the confluence of political, socio-economic, and geostrategic factors provided a window of opportunity for OCGs to empower as rulers in this borderland. Through the control of illegal paths also known as *trochas*, the regulation of flows of people and goods across the border, and the control over labor market and migrant population, organized crime brings to bear in the (re)production and managing of the border.

The paper speaks to different literatures in the realms of criminology and both security and border studies. We make a case for bordering practices as a realm of non-state governance and show that criminal organizations engage in bordering practices in a way that goes beyond the mere opportunistic or exploitative use of borders. Based on the study of the Colombia–Venezuela borderlands, we demonstrate how recent political developments provided conditions and incentives for criminal actors to actively partake in bordering practices, notably border management and control of the immigrant population. Bridging theoretical contributions from border studies with recent debates on governance and crime, our analysis shades light on the role that illegal actors play in the process of construction and deconstruction of national borders.

The rest of the paper is structured as follows: we first introduce the conceptual tenets of the analysis. Second, we describe the research strategy, case selection and data sources and analysis. Third, we contextualize the trajectories of conflict, violence, and crime in the Colombian-Venezuelan borderlands. Fourth, in the main section of the paper, we analyze the linkages between organized crime and bordering practices. Finally, we sum up the findings and discuss their implications in the understanding of the linkages between organized crime and borderlands’ spatiality.

### Border, borderlands, and bordering practices

Borders are institutions (Newman 2011; Paasi 1998). They enact a stable set of rules for human behavior and interaction (North 1990) and fulfill a set of functions. The defining nature of any border is the establishment of a division between spaces in political, socio-economic, spatial, and symbolic terms. Through border control, states define and regulate what people and goods have legitimate access to their territory (Andreas 2003). As a spatial division, one of the main functions of the border is to protect or ‘to act as a barrier’ (Newman 2011: 15) against actors and goods deemed as threats or harmful. While traditional military threats and economic regulation were the main concern for border protection, the state’s priorities regarding border security have shifted towards policing and law enforcement (Andreas 2003). The target of policing is to avoid the territorial access of a plethora of non-state actors, ranging from drug traffickers, terrorists, and smugglers to unauthorized migrants. Borders also regulate trans-border circulation, defined as the ‘in and out’ movement between different state territories (Newman 2011).

However, borders are also spaces of interaction and contestation. One of the ‘paradoxes of borders’ is that they divide and unite at the same time (Baud and van Schendel 2005). Another facet connatural to borders is contestation. Several actors that aim to manipulate, take advantage or circumvent border control persistently challenge borders and the regulation that they embody. The complex and heterogeneous interplay among rules, practices, and actors is what shapes borders and borderlands. Borders are therefore a socio-territorial construction resulting from the legal and geopolitical discourses of states, on the one hand, and the action of border societies, on the other. In what is a co-constitutive relationship, the very existence of the border crosses and affects the character and dynamics of the areas affected by its presence. The impact of the border goes beyond the boundary as such and extends into the territories whose nature and conditions are shaped by the nature of border itself (Zartman 2010: 5). The literature refers to these areas with the concept of borderlands (Baud and van Schendel 2005; Idler 2019; Newman 2011; Zartman 2010). Idler (2019) argues that the relationship with the central state and transnationality are key aspects of borderlands.

Like any institution, borders are constantly changing (Paasi 1998). While some borders are enhanced, others wither away. Processes of regional integration imply a reduction or abolition of border controls. For instance, the creation of the ‘Schengen border-free zone’ implied the removal of border controls among most of the member countries of the European Union. In contrast, security measures in the aftermath of 9/11 led to the strengthening and reclosing of borders. The notion of ‘bordering practices’ refers to the actions involved in creating, sustaining, and modifying borders between states (Parker and Adler-Nissen 2012: 776). Bordering practices encompass a wide range of deeds performed by both the state and non-state actors. According to Parker and Adler-Nissen (2012), when it comes to order-making processes in borderlands, bordering practices are more important than the existence of the border itself.

The literature on borderlands has drawn attention to the role of societal actors in producing and sustaining borders. The state interacts with different non-state actors, such as international organizations or multinational companies, to define and implement border policies (Gavrilis 2008). In the framework of debates regarding the vernacularization of borders (Cooper et al. 2016), Rumford (2014) coined the term ‘border work’ to define ‘the activity of ordinary people leading to the construction or dissolution of borders, and driven by their own ‘grassroots’ agendas rather than those of the state” (p. 23). Although the conceptualization of Rumford expands beyond state borders to encompass any spatial scale, the term has been used to analyze the role of borderland communities and NGOs in bordering practices (Lamb 2014; Laurie et al. 2015).

### Organized crime, criminal governance, and borders

Organized crime is a multifaceted phenomenon. Hence, the concept remains fragmented and vague (von Lampe 2016). A way to make sense of such complex phenomenon is looking into the activities of OCGs (Sergi 2014; Varese 2017), focusing on the actions (crimes) of criminal groups and the way they are carried out (Sergi 2017). The role of power and the capacity to exert social regulation has been crucial in the theorization of the activities of organized crime. The literature identifies one set of market-oriented activities involving the production and trade of illegal goods (von Lampe 2016; Varese 2017). For instance, Block (1983) refers to the groups that operate exclusively in the realm of illicit economies as enterprise syndicates. The main purpose of these groups is the provision of goods and services prohibited or highly regulated by the state. In the realm of illegal markets, there is a high level of specialization, with some groups focused on the production of goods and services, and others specializing in the trading of illegal products (Shortland and Varese 2016).

Another set of activities is defined by the engagement of OCGs in the provision of protection and their capacity to regulate conflicts within markets or among competitors. In this regard, Block (1983) identifies a second type of criminal syndicates under the label of power syndicates. Unlike enterprise syndicates, the strong point of power syndicate is extorsion and the ability to coerce standing as enforcers and as violence specialists. For instance, the role of coercion has been central to many theorizations of organized crime. Gambetta (1993) argues that the landmark of mafias is the provision of private protection and its distinctive function of securing transactions. 

In a similar vein, Sergi (2017) posits that the capacity to govern is what distinguishes mafias as a subgroup within the broader category of organized crime. More recently, based on the definition of economic governance as “the set of rules and norms that regulate exchange” (p. 44), Varese (2017) has claimed that organized crime is a form of governance and part of a continuum that includes criminal organizations, insurgent groups, and the state. Hence, criminal organizations are conceived as competing actors in the aspiration of governing exchanges. In this paper, we understand organized crime as a phenomenon that encompasses both illegal enterprises and governance activities. Following Shortland and Varese (2016), this case study presents the engagement of OCGs in three types of activities: production of illegal goods, trading of illegal goods, and finally, governance.

Regarding the last one, the concept of criminal governance has emerged as a way to underscore the role of criminal organizations as de facto rulers and their capacity to shape social order (Arias 2017; Barnes 2017; Lessing 2020). The existence of criminal governance does not imply the absence or suspension of state governance. On the contrary, as scholars have extensively documented it implies complex interactions and exchanges in which the state and OCGs are not always at loggerheads (Arias 2006; Auyero 2007; Snyder and Duran-Martinez 2009; Sciarrone and Storti 2014; Dewey 2015; Denyer Willis 2015; Dagnes et al. 2018; Lessing 2018; Sobering and Auyero 2019).

Drawing upon this notion, we argue that criminal actors are part of the governing authorities in the Colombian-Venezuelan borderlands. They do not only partake in the production and trade of illegal goods but govern over borderland communities in a broad sense. They constitute local authorities setting rules while providing public goods and services. Against this backdrop, migration control and customs (public goods) are shaped and cooptated by OCGs. They regulate human mobility and govern immigrants’ and borderland communities’ lives. Therefore, the activities in which they are engaged fit within those classified as bordering practices.

### Empirical strategy

The analysis is methodologically arranged as a case study. Given that the border area encompasses more than 2.200 km2, our study focuses on the department of Norte de Santander (Colombia). We chose this area because the most important border crossing points are located there. Additionally, it is at the heart of the local economy as the axis formed by the cities of Cucuta in Colombia and San Antonio in Venezuela constitutes the most relevant urban system in the borderland. Norte de Santander has been the entry point for 94 % of Venezuelan migrants traveling to Colombia by land (OCHA 2018). Likewise, this department concentrates the second largest population of Venezuelans in Colombia (OCHA 2018; Migración Colombia 2020).

The research draws on semi-structured interviews and direct observation conducted in 2019 and 2020. The interviews —15 in total— were conducted in Cúcuta, San Antonio, and Villa del Rosario. Following a purposive sampling technique,^2^ they feature border-crossers, local government officials, journalists, Cucuta’s Police Department members, non-profit organizations’, and humanitarian workers. The interviews were analyzed using pattern coding (Saldaña 2009) and the data elicited was triangulated with other sources of information such as local and national newspapers, reports of NGOs, and secondary literature. Regarding direct observation, it was done in the urban area of Cucuta, and the zone next to the border, specifically the area known as “La Parada”.^3^ Aditionally, the study relies on statistics from official sources and reports by humanitarian organizations.

### Crime and conflict in Colombia–Venezuela borderlands

The border between Colombia and Venezuela has played a crucial role in the Colombian armed conflict and the illegal economies that fuel war and crime. While the presence of armed actors in this area goes back to the decades of 1970 and 1980, it is possible to identify three periods in the recent history regarding war and organized crime. The first period known as the paramilitary offensive goes from 1999 to 2004. It sparked massive displacement of communities and members of leftist guerrillas to Venezuela. The second one is comprised of different bilateral crises amid the alleged support of Colombian guerrillas by the Venezuelan government and goes from 2008 to 2013. During this period, Colombian guerrillas were debilitated through peace and military efforts and pushed to Venezuela as a rear-guard position. The third period, in which this paper focuses, goes from 2015 until date. It starts with the Venezuelan government’s decision of closing the border amid the massive exodus of Venezuelans that sparked a migratory and humanitarian crisis as well as the rupture of bilateral relations with Colombia.

Since the early 2000s, Venezuela’s security situation has deteriorated in the context of an acute economic crisis, authoritarian practices of the Bolivarian regime, and political polarization. Along the increase of state violence (Ávila 2019), the country has seen the sprout of a myriad of criminal organizations (*pranes*) and paramilitary groups (*colectivos*), which gained control over the illicit markets such as drug trafficking, territories, and populations, chiefly in urban slums and marginalized areas. With a homicide rate of 60,3 per 100.000 inhabitants in 2019 (Observatorio Venezolano de Violencia 2020), Venezuela turned into the most violent country in the world. In the last seven years (2013–2020), the exodus of Venezuelan nationals fleeing political and economic crisis in their country has reached similar proportions to those provoked by the civil war in Syria (Fieser 2019). As the security situation in Colombia–Venezuela borderlands further deteriorates, their diplomatic relationship has followed the same path leading to a proxy war setting. According to Groh (2019) proxy support includes the direct or indirect use of non-state and parastate groups to carry out militarized intimidation or control territory in order to exert influence or achieve specific security or political outcomes. The relation between the state and non–state armed actors (proxy) is one of patron–agent; the state either directs proxy’s actions or partakes on them. Let us to further elaborate on this point.

On the one hand, Colombia’s government has denounced several times the existence of a coordinated strategy and support between the Venezuelan regime and Colombian rebel groups, namely the National Liberation Army (ELN) and rearming dissident factions of the former Colombian Revolutionary Armed Forces (FARC) (Reuters 2019). Likewise, several social organizations and journalists have reported on the constant activity of Colombian armed groups in at least seventeen states of Venezuela and mainly in the borderland ones of Zulia, Táchira and Bolivar. According to local reports, along its control over gold mines and other economic assets, the ELN guerrilla recruits new members and has training camps in these areas (Fundaredes 2018). For Colombia, such support from the Venezuelan government to Colombian insurgencies is a hostile act and a direct threat to its national security.

On the other hand, Colombia is the main geopolitical platform against the Venezuelan regime. As of 2020, the government of Colombia has committed to a “de facto” political transition agenda by recognizing the self–proclaimed president of Venezuela —Juan Guaidó— as the legitimate president of the country, while Nicolas Maduro keeps having command and control over the state apparatus. Colombia has played a central role in regional and hemispheric initiatives to push for a democratic transition in Venezuela, such as the Lima Group. In early 2019, Colombia’s government organized a concert and humanitarian action in the border area. The occasion was used to overtly express support to Guaidó, who gave a speech during this activity. In what later became a scandal, it was revealed that local Colombian authorities facilitated the irregular crossing of Guaidó through the participation of members of “Los Rastrojos”, an organized crime group of paramilitary ethos (The Guardian 2019). One hardly could find a better instance to illustrate the linkages between the state and criminal actors and the overlap of legal and illegal spheres in the borderland than this one.

The current relationship between Colombia and Venezuela can be defined as one of enmity, given that both countries blame each other for advancing hostile strategies to destabilize its counterpart. This has contributed to further aggravate the security and humanitarian crisis in the borderland as no security, immigration or border bilateral policy can be neither coordinated nor developed. Against this background, the region has provided a context favorable for the further flourishing of a plethora of non-state armed groups. The following table offers a list of some of the most relevant criminal groups. They encompass OCGs as well as hybrids —insurgencies involved in organized crime activities. Given the fragmentation, the rapid pace of change of these groups, and the diversity of organizational structures, the list may not reflect the current constellation of armed actors (Table 1).

**Table 1:** Non-state armed groups in the Colombian-Venezuelan borderlands

Name	Type	Illegal Activities
National Liberation Army ELN	Leftist insurgency	War Economy (Includes: large scale drug trafficking control, illegal mining, public budgets cooptation, immigration control)
Popular Liberation Army EPL or ‘Pelusos’	Former leftist insurgency that turned into an organized-crime like group	Large scale drug trafficking, immigration control; gas smuggling
FARC dissidence	Member of the former front 33 of the FARC	Large scale drug trafficking
Los Rastrojos	OCG rooted in former paramilitary militias	Large scale drug trafficking, illegal paths control, irregular immigrants taxation, extortion, gas smuggling
Tren de Aragua	OCG of Venezuelan origin	Illegal paths control, irregular immigrants taxations.
Clan del Golfo	OCG rooted in former paramilitary militias, mostly operating in the area Cucuta.	Drug trafficking and distribution, gas smuggling.
La Linea	OCG	Illegal paths control, irregular immigrants taxation, human trafficking
La Frontera	OCG of Venezuelan origin	Illegal paths control, irregular immigrants taxation, human trafficking
Los Diablos, Los Canelones, and Los Cebolleros	Small OCGs mostly operating in the area of Cucuta	Small scale drug distribution, extortion, contract killings
“Botas de Caucho”	OCG. According to some accounts, this group is another denomination for members of ELN.	Allegedly under the operative umbrella of ELN

Source: Authors’ elaboration based on interviews, officials and NGO’s reports

### Enforcing and exploiting the border: Illegal paths and criminal governance

Smuggling has been a connatural activity to many borderlands (Andreas 2000; Sadikki 2017), and Norte de Santander’s borderland is not the exception. Since the second half of the twentieth century, smuggling of goods such as fuel gas and electronics was regarded as a common activity that has effectively merged with the formal economy. This moral economy is not only the result of sociocultural practices of border–crossing regarded as a legitimate way of making a living but also derives from the political and economic denial of the region by political elites from Bogotá and Caracas (Idler 2019). Illegal paths or *trochas* have been the spatial mechanism enabling this cross-border activity and can be regarded as a corollary of the “border work” of communities.

However, amid the war and criminal activities, the illegal paths have also been used by different armed actors. While one can argue that bordering practices by armed groups were not a new phenomenon in the area, the current migratory and humanitarian crisis and the closure of the border in 2015 contributed further to strengthen the power of these actors over borderlands. Although the closure was part of a strategy of organized crime control by the Venezuelan state, it had a marginal impact on deterring irregular immigration and criminal activities. Instead, it created a window of opportunity for organized crime to diversify their illegal profits, extend its control over human mobility, and set new mechanisms through which state officials engage in collusion arrangements with OCGs creating a situation of border control paradox (Mantilla 2019). In the case under analysis, the further border enforcement over binational communities as a political and military artifact changed the relationship between OCGs and space benefiting OCGs in at least three ways.

#### Turf over drugs

First, the border closure produced a shock in illegal markets and spatial practices since the control of the paths for traditional smuggling and the informal crossing became a major source of illegal profits. With about 40,000 people crossing every day —in between pendular immigrants and people trying to flee Venezuela— the closure of international ports of entry spilled thousands of people to illegal paths and into the hands of those who control them, altering the current balance of power among organized crime and state authorities. As a local journalist accounts: “*Smuggling at the border is not anymore just about the gasoline. Everything is crossing through the border, which actually makes the control of an illegal path much more profitable than selling drugs or other illegal business. Every day, every minute people are crossing and so someone is making money”*.^4^

Despite the instability that is supposed to characterize illegal economies and smuggling settings, the tolls that OCGs charge to allow border crossing are fixed and vary according to the goods and people to be smuggled. Thus, an irregular immigrant pays a fixed toll for crossing while a truck with licit goods trade illegally, such as medicines or chemical supplies, pays a different one. This scheme of prices matches findings reported in other contexts where informal institutions display differential regulatory nodes according to the type of good (licit/illicit) smuggled (Gallien 2019). As in any other market, prices react to changes in demand. Thus, after the Colombia government decided to enforce closure measures at the border in the face of the COVID-19 public health crisis, the price that OCGs were charging to people to use the *trochas* spiked in almost five hundred percent (El Tiempo 2020).

#### Assisted travel and micropolitics of informality

Second, besides contributing to further turning the illegal paths into highly regarded assets, the closure of 2015 paved the way for the configuration of both an informal sphere of control of transnational migratory flows and an expanded portfolio of illegal and legal markets. According to Antillano, Zublillaga, Sánchez & Ortíz (Forthcoming), people relying on illegal paths for the sake of immigrating to Colombia are those who do not have a valid passport or a migratory card, those who do not have enough time to make the lagging lines to get their passports stamped by Colombian migration authorities, or those that were previously deported by the Colombian immigration authority. Migrants’ precariousness makes them dependent on the services offered by OCGs as well as particularly vulnerable to different forms of victimization.

Furthermore, OCGs play a key role for people trying to flee Venezuela given that they provide access to services and connections that are crucial for the immigrants. The services feature the crossing to the Colombian side and travel arrangements from this point to other cities in Colombia or countries in the region. A bus terminal located next to the “Simón Bolívar” bridge, the main border-crossing point, is the next stage for many migrants after leaving Venezuela. Many of our informants commented about the links between OCGs and travel agencies located in the area. OCGs also provide the means of connection to other illegal economies such as sex trade or coca crops related employments. As an illicit market, smuggling does not always rely on violence. Rather, it depends on the broader political and social context wherein the criminal group operates more than the nature and the structure of the group itself (Andreas and Wallman 2009; Sanchez 2015). In this sense, the variation on the use of coercion makes difficult to set a clear-cut division between the provision of services demanded by immigrants and human trafficking. Although further investigation is required, the evidence collected suggests that OCGs are involved in both activities. Some participants talked about the role of OCGs as service providers and facilitators, whilst others point out that some immigrants are brought to the country in the frame of human trafficking networks as victims of different forms of exploitation.

Likewise, OCGs became de facto rulers in the neighborhoods adjacent to the border. They organize the micro-politics of informality and deliver street justice in places such as “*La Parada*”, where immigrants try to have access to humanitarian aid, transportation services, street vending entrepreneurship, or immigration and work permissions. While OCGs perform the role of service providers and social authorities, they also make use of different repertoires of violence and terror, such as beheadings or dismemberments. This is the case of “La Frontera,” an OCG responsible for multiple massacres between 2016 and 2018. In one of the incidents that occurred in January of 2018, body parts of four victims were spread on one of the *trochas* connecting the two countries (La Opinión 2018a). In the investigations leading to set the suspected responsibility of this group, more than one hundred Venezuelan and Colombian ID cards were found in a warehouse revealing the extent to which this group had control of irregular immigrants who were previously reported as missing or who were surrendered to human trafficking networks (La Opinión 2018b).

Engaging in the activities described above, OCGs have become the ultimate customs and migratory authorities, defining the circumstances of place, means, and time in which the border can be crossed or not. Furthermore, they perform border governance in ways that assure reliability to those using smuggling services. Following Idler (2019) criminal governance at the border resembles a social contract–like relationship in which OCGs provide public goods and services and define the rules of appropriate behavior while neglected citizens recognize their shadow authority.

#### Systematic collusion

Third and lastly, the border closure contributed to strengthen collusion mechanisms between the state and OCGs. The relationship between the state and illegality is a complex one in borderland contexts. For Goodhand (2013), state presence in these contexts is contingent, contested, and changing. In contrast to previous research that addresses criminality in Colombian borderlands as the consequence of state fragility and its incapacity to deliver effective security (Idler 2019; Castrillón and Valencia 2019; Villa and Souza 2019), we build on the assumption that state presence cannot simply be equated with law and order. As previous literature has noted, organized crime requires the “studied ignorance or tacit consent”(Schneider and Schneider 1999) of the state to work. Licit and illicit practices coexist and are imbricated in manifold ways. More so, illicit practices are often part of the processes of state-making and border control (Abraham and Van Schendel 2005; Goodhand 2013; Gallien 2019).

The strategic interactions between border enforcers and unauthorized border crossers that Andreas (2000) calls “border games” imply that state authorities and OCGs often collaborate through the means of corruption. This is a widespread practice in Norte de Santander where, for years, the overwhelming power of paramilitaries and guerillas left no choice for law enforcement units rather than comply with de facto *omerta*. However, mechanisms of collusion between the state and OCGs have developed in such ways that, for many residents and participants in illegal economies, state security forces are just, or at least behave as, another OCG. Regarding traditional bribery, corruption schemes work through monthly payments depending on the OCG and the rank of state agents. For instance, in the municipality of Puerto Santander, an agent cooperating with “Los Rastrojos” receives from 80 to 100 dollars per month, while higher ranks can receive between 200 and 300 dollars. According to both police officers and their families, it is not even an option to say no. A brother in law of a Police captain deployed in one of the border checkpoints said “*the police here is still afraid of the armed groups, so they limit their job to a passive and blind eye one in the context of generalized extortion. My brother in law is a couple of years away from retirement; he doesn’t want problems or unnecessary risks at this point so what else he can do?”*^5^

Nevertheless, only a couple of weeks after this interview, counterintelligence and anti-corruption units captured the police intendant of Puerto Santander for being an active member of *Los Rastrojos*. According to the indictment, he was the second in the command line of this OCG and has been an active member of this group for the last six years (La Opinión 2020). Less than a month after this incident, Venezuelan security forces captured the police chief of Táchira while she was transporting ammunition, pamphlets and war material for *Los Rastrojos* (La Prensa Táchira 2020). These incidents suggest that the relation between OCGs and the state goes beyond mere intimidation and bribery and that schemes are more similar to state sponsor protection rackets (Snyder and Duran-Martinez 2009) or that state capture (Garay-Salamanca and Salcedo-Albarán 2012) may be at play.

Other evidence highlights the fact that state officials directly victimize vulnerable immigrants. Reports about Colombian migration officials running a scheme of extortion in exchange for easing the migratory process are common in the soup kitchens that philanthropists and volunteers’ efforts have brought together at immigrant shelters at the border. Presumably, immigration officials retain Venezuelan passports and then ask for a money ransom to give them back. These forms of victimization are gender informed. Thus, in the case of women, passports can be retained for the sake of sexual favors. It is important to note that, in the context of a migratory crisis and the vulnerability of people fleeing Venezuela, passports are the most valuable and useful good someone can hold and, therefore, a mechanism of power and control. Victimization infringed by state officials may explain the gap between locals’ perceptions of the *trochas* vis–á–vis state narratives of dangerous criminal groups controlling these illegal paths. Similarly, for gasoline smugglers the police Fiscal and Customs Police (Polfa in Spanish) is regarded as just another OCG. When asked about the interactions with the police, one of their most recognized leaders vehemently claimed: *“About the police, I can only say that they are just another group. They steal from us in our face, just like that, straight forward. They confiscate ten pimpinas*^6^*from us but they only report two. The complicity between criminal bands and the police is pretty obvious from what you can see that is going on”.*^7^

The account of how locals from the Norte de Santander feel about the border as an artifact imposed by OCGs and by the state fit into what Abraham and Van Schendel (2005) describe as a gap in between behaviors and identity when talking about criminality across borders. The fact that OCGs and the state have similar behaviors to the eyes of the community highlights that the state authority is just one form of political authority among others in the borderlands. The variety of social agents involved in bordering practices, the diversity of the interactions between this variety of actors, and the institutional effects of the parallel set of norms serve to outline the complexity of the relationship between the state, organized crime, and space in the borderlands.

### Bordering practices and organized crime beyond the *trochas*

As mentioned above, the precariousness of the migrants makes them vulnerable to several forms of exploitation and victimization. Criminal organizations leverage the availability of cheap labor; they have recruited Venezuelan migrants as part of their armed structures or integrated them into illegal markets such as drug production and sexual exploitation (Fundaredes 2018; La Opinión 2019; OCHA 2018).

This is the case of the coca crops and coca laboratories. The Catatumbo region, which ranks as the third with the largest number of hectares of coca cultivation in Colombia (UNODC 2019), is part of the Colombia-Venezuela borderlands. It is estimated that around 25,000 Venezuelans are in the Catatumbo (OCHA 2019). Some informants recounted that in the municipality of Tibu —which has a high number of hectares of coca cultivation in the region— many of the “*raspachines*”^8^ are Venezuelan and noted that the composition of the workforce in coca cultivation in some villages of this municipality went through a transformation over the last years with Venezuelans currently making up at least 70% of the people working on the crops. An officer of a local NGO^9^ commented that people working in coca leaf production could earn up to U$120 dollars per week. Upon arrival in the territory, migrants must abide by the rules and authority of the incumbent OCG. In some cases, they must carry a letter of recommendation to prove that they have “authorization” to live there or must be recommended by a local person to access to the community. In the face of conflicts between settlers and immigrants, OCGs also intervene to solve conflicts and disputes.

Since the Colombian state has no control over the population entering the country through the *trochas*, local dwellers and humanitarian organizations report that groups such as EPL and ELN have enforced their own mechanisms to manage and control immigrants. According to OCHA (2018), ELN issues a sort of migration permit either when people are crossing through illegal paths or when migrants arrive in communities under their control. The license serves as a means to control immigrants’ mobility and access to job market. Similarly, some of our informants note that this group exerts the same practice in the Catatumbo region.^10^ In this case, the permit is valid for sixty days and allows the migrant to stay in the region and work; its renewal depends on an assessment of the behavior of the migrant and their compliance with criminal governance rules.

## Discussion and conclusions

Traditional state-centric perspectives on crime, borders, and governance have overshadowed the complexities of the relationship between organized crime and borderlands (Abraham and Van Schendel 2005). Considering this, the article has presented an analysis of the engagement of organized crime on bordering practices and the co-production of borders in the case of the Colombia-Venezuela borderlands.

Contrary to common assumptions on organized crime bypassing or circumventing state borders, our study showed that criminal organizations also seek to set norms and regulate the cross-border flux of goods and people; willing to enforce the border for their own benefit if the strategic environment provides the opportunity to do so. Similar to other cases (Massey 2017; Tinti and Reitano 2017), rather than deterring organized crime activities, the closure of the border by the Venezuelan government created new opportunities and assets for OCGs. Namely, it enhanced criminal organizations such as Colombian guerrillas, OCGs of paramilitary ethos, and bi-national gangs to further engage and control human and goods smuggling operations, border management, and social control over the migrant population.

As shown by previous research, interactions among OCGs and between these and the state can follow different patterns ranging from enmity, to rivalry and cooperation (Idler 2019:40). In short, the relationship between OCGs and borderlands should not be taken for granted as traditional national security approaches suggest. Bordering practices in the context of illicitness shape local economies and institutions considered rightful by locals (Gallien 2019; Rolandsen 2019).

In addition, organized crime activities in the borderlands exceed conventional definitions focused on the provision of illegal goods, services, and predatory crimes. Rather OCGs also constitute the ultimate customs and migratory authorities. Particularly, in spatial terms, organized crime power goes beyond the management of the clandestine paths and extends into other levels of the social order in the borderland. For many Venezuelan migrants, entering Colombia is tantamount to entering a realm where spatial practices, social regulation, and their own life are shaped not by the rules of the Colombian state but by OCGs. Bordering practices by organized crime shape the borderland and the life of its inhabitants.

This case illustrates the complexities of border politics. Borders are not the result of the command of the state, but instead of the practices and interaction of multiple actors, OCGs among them. In this regard, the analysis sheds light on how spatial particularities of the borderlands, and the actions of OCGs have profound consequences on the management and (re)shaping of the border. Thus, similar to what literature on border studies have described in the case of other societal groups, it is possible to argue that OCGs also engage in ‘border work’ (Rumford 2014) and perform bordering practices. Colombia-Venezuela borderlands are shaped by complex interactions between the state, local communities, migrant population, and organized crime. One of the main limitations of this paper and an avenue of future research is to assess how OCGs build legitimacy over negotiations with borderland communities. In addition, although this borderland is a place of despair, it is also a source of hope for thousands who are able to perform social resilience. Further research should look into what kind of life and agency is possible for those under criminal governance.

Bordering practices is, hence, another facet of criminal governance in transnational contexts and should be considered as a set of practices to further document and study within organized crime literature. If we accept the idea that the activities of organized crime are a form of governance (Varese 2017), or that OCGs can decide whether to exert criminal governance or not (Lessing 2020), then we should consider competitive border–making as an area of dispute, cooperation, and exploitation between OCGs and the state. Adding to concepts coined to express the particularities of security in borderlands, such as *border effect* (Idler 2019), we think that bordering practices offer an untapped analytical potential to understand how and why OCGs get involved in the provision of public goods, the enforcement of rules, and co-constitutive linkages to space.

## Footnotes

1 During the last five years the border has been reopened in several occasions and for short periods.

2 “Purposive sampling refers to strategies in which the researcher exercises his or her judgment about who will provide the best perspective on the phenomenon of interest, and then intentionally invites those specific perspectives into the study” (Abrams 2010, p. 538).

3 There the authors visited humanitarian kitchen soups. They also crossed the border through the “Simón Bolívar” crossing point.

4 Interview with regional journalist, Cúcuta, July 2019.

5 Interview local resident, Cúcuta, July 2019.

6 Pimpinas are recipients in which smugglers carry gasoline. They are equitable to several gallons and the reason why gasoline smugglers identify themselves as “pimpineros”.

7 Interview with gasoline union affiliate, Cúcuta, January 2020.

8 This is the name given to workers that harvest (scrape) coca leaf.

9 Interview officer of humanitarian NGO, Cúcuta, August 2019.

10 Interview with a grass-roots organization’s leader, Cúcuta, August 2019.

## References

[CR1] Abraham I, Van Schendel W (2005). Illicit flows and criminal things states, borders, and the other side of globalization.

[CR2] Abrams LS (2010). Sampling ‘hard to reach’ populations in qualitative research. Qualitative Social Work: Research and Practice.

[CR3] ACNUR (2019) Situación en Venezuela. Available at: https://www.acnurorg/situacion-en-venezuela. Accessed 20/04/2020

[CR4] Andreas P (2000). Border games: policing the US-Mexico divide.

[CR5] Andreas P (2003). Redrawing the line: Borders and security in the twenty-first century. Int Secur.

[CR6] Andreas P (2013). Smuggler nation how illicit trade made america.

[CR7] Andreas P, Wallman J (2009). Illicit markets and violence: what is the relationship?. Crime Law Soc Chang.

[CR8] Arias E (2006). The dynamics of criminal governance: networks and social order in Rio de Janeiro. J Lat Am Stud.

[CR9] Arias E (2017). Criminal enterprises and governance in Latin America and the Caribbean.

[CR10] Auyero J (2007). Routine politics and violence in Argentina.

[CR11] Ávila K (2019) Una masacre por goteo: Venezuela y la violencia institucional. Nueva Sociedad*.* Available at: https://nuso.org/articulo/venezuela-maduro-represion-izquierda. Accessed 13/03/2020

[CR12] Barnes, N (2017) Criminal politics: an integrated approach to the study of organized crime, politics, and violence. Perspectives on Politics American Political Science Association Special Section Articles, Vol. 15–4

[CR13] Baud M, van Schendel W (2005). Toward a comparative history of borderlands. Journal of World History.

[CR14] Block A (1983). East side, west side: organizing crime in New York 1930–1950.

[CR15] Castrillón J, Valencia J (2019) Reconfiguración de la frontera Norte de Santander-Táchira: Ilegalidad, Crimen Organizado y Corrupción. Revista Opera 24:157–177

[CR16] Cooper A, Perkins C, Rumford C, Jones R, Johnson C (2016). The Vernacularization of Borders. Placing the border in everyday life.

[CR17] Dagnes J, Donatiello D, Moiso V, Pellegrino D, Sciarrone R, Storti L (2018). Mafia infiltration, public administration and local institutions: a comparative study in northern Italy. Eur J Criminol.

[CR18] Denyer Willis, G., 1979-. (2015). The killing consensus: police, organized crime, and the regulation of life and death in urban Brazil. Oakland, California: University of California Press

[CR19] Dewey M (2015). El Orden Clandestino: Política, Fuerzas de Seguridad y Mercados Ilegales en Argentina.

[CR20] El Tiempo (2020) Grupos Criminales cobran hasta $100.000 por dar paso en la frontera. Available at: https://www.eltiempo.com/colombia/otras-ciudades/cierre-de-frontera-con-venezuela-panorama-de-medidas-por-coronavirus-en-norte-de-santander-474356. Accessed 22/04/2020

[CR21] Fieser E (2019) Venezuela Exodus is as big as Sirya’s. Yet Got 1% of the Aid. Available at: https://www.bloomberg.com/news/articles/2019-09-20/venezuela-exodus-as-big-as-syria-s-got-1-5-of-the-aid-chart. Accessed 20/01/2020

[CR22] Fundaredes (2018) Informe Anual Fundaredes: 2018. Available at: https://www.fundaredes.org/2019/04/12/informe-fundaredes-2018/. Accessed 15/01/2020

[CR23] Gallien M (2019). Informal institutions and the regulation of smuggling in North Africa. Perspectives on Politics.

[CR24] Gambetta D (1993). The Sicilian mafia: the business of private protection.

[CR25] Garay-Salamanca LJ, Salcedo-Albarán E (2012). Institutional impact of criminal networks in Colombia and Mexico. Crime Law and Social Change.

[CR26] Gavrilis G (2008). The dynamics of interstate boundaries.

[CR27] Goodhand J (2005) Frontiers and wars: the opium economy in Afghanistan. J Agrar Chang 5(2):191–216

[CR28] Goodhand J (2013) The view from the border. In: Korf B, Raeymaekers T (eds) Violence on the margins: state, conflict, and borderlands. Palgrave Macmillan, New York

[CR29] Groh T (2019) Proxy war: the least bad option. Stanford University Press, Stanford CA

[CR30] Idler A (2019). Borderland battles: violence, crime, and governance at the edges of Colombia’s war.

[CR31] La Opinión (2018a) Las huellas delataron a los homicidas de cuatro venezolanos en La Parada. Available at: https://www.laopinion.com.co/judicial/las-huellas-delataron-los-homicidas-de-cuatro-venezolanos-en-la-parada-153504#OP. Accessed 20/03/2020

[CR32] La Opinión (2018b) La Parada: una masacre anunciada? Available at: https://www.laopinion.com.co/judicial/la-parada-una-masacre-anunciada-147534. Accessed 13/03/2020

[CR33] La Opinión (2019) Narcocultivos en Tibú, el imprevisto destino de migrantes venezolanos. Available at: https://www.laopinion.com.co/frontera/narcocultivos-en-tibu-el-imprevisto-destino-de-migrantes-venezolanos-171238#OP. Accessed 28/03/2020

[CR34] La Opinión (2020) Capturan a Policía en Cúcuta señalado de ser cabecilla de “Los Rastrojos”. Available at: https://www.laopinion.com.co/judicial/capturan-policia-en-cucuta-senalado-de-ser-cabecilla-de-los-rastrojos-191143#OP. Accessed 12/03/2020

[CR35] La Prensa Táchira (2020) Detienen a mujer policía con 630 municiones de guerra. Available at: https://laprensatachira.com/nota/7679/2020/02/detienen-a-mujer-policia-con-630-municiones-de-guerra. Accessed 15/07/2020

[CR36] Lamb V (2014). “Where is the border?” villagers, environmental consultants and the ‘work’ of the Thai–Burma border. Polit Geogr.

[CR37] Laurie N, Richardson D, Poudel M, Townsend J (2015). Post-trafficking bordering practices: perverse co-production, marking and stretching borders. Polit Geogr.

[CR38] Lessing B (2018) Making peace in drug wars: crackdowns and cartels in Latin America. Cambridge University Press, New York

[CR39] Lessing B (2020). Conceptualizing Criminal Governance. Conceptualizing Criminal Governance.

[CR40] Mantilla J (2019) The Border control paradox in Venezuela. Available at: https://www.law.ox.ac.uk/research-subject-groups/centre-criminology/centreborder-criminologies/blog/2019/12/border-control. Accessed 23/02/2020

[CR41] Massey D (2017) The counterproductive consequences of border enforcement. The Cato Journal Cato Institute 37(3)

[CR42] Migración Colombia (2020) Más de 1 millón 825 mil venezolanos estarían radicados en Colombia. Available at: https://www.migracioncolombia.gov.co/noticias/265-abril-2020/mas-de-1-millon-825-mil-venezolanos-estarian-radicados-en-colombia. Accessed 12/05/2020

[CR43] Newman D (2011). On borders and power: a theoretical framework. Journal of Borderlands Studies.

[CR44] North D (1990). Institutions, institutional change, and economic performance.

[CR45] Observatorio Venezolano de Violencia (2020) Informe Anual. Available at: https://observatoriodeviolencia.org.ve/news/informe-anual-de-violencia-2019/. Accessed 12/03/2020

[CR46] OCHA (2018) Briefing Departamental. Norte de Santander. Available at: https://www.humanitarianresponse.info/es/operations/colombia/document/brieffing-norte-de-santander. Accessed 24/07/2020

[CR47] OCHA (2019) Boletín Humanitario Febrero 2019. Available at: https://www.humanitarianresponse.info/en/operations/colombia/document/bolet%C3%ADn-humanitario-febrero-2019. Accessed 20/04/2020

[CR48] Paasi A (1998). Boundaries as social processes: territoriality in the world of flows. Geopolitics.

[CR49] Parker N, Adler-Nissen R (2012). Picking and choosing the “sovereign” border: a theory of changing state bordering practices. Geopolitics.

[CR50] Payan, T. (2006) The three U.S.-mexico border wars: Drugs, immigration, and homeland security. Westport, Conn: Praeger Security International

[CR51] Reuters (2019) Colombia’s Duque tells UN the Dossier proves Maduros supports terrorists. Available at: https://www.reuters.com/article/us-venezuela-politics-colombia/colombias-duque-tells-un-that-dossier-proves-maduro-supports-terrorists-idUSKBN1WA2XH. Accessed 10/03/2020

[CR52] Rolandsen O (2019). Trade, peace building and hybrid governance in the South-Sudan Sudan borderlands. Confl Secur Dev.

[CR53] Rumford C (2014). Cosmopolitan Borders.

[CR54] Saddiki S (2017) World of walls: the structure, roles and effectiveness of separation barriers. Open Book Publishers

[CR55] Saldaña J (2009). The coding manual for qualitative researchers.

[CR56] Salehyan, I (2011) Rebels without borders: Transnational Insurgencies in World Politics Ithaca: Cornell University Press

[CR57] Sanchez G (2015). Human smuggling and border crossings.

[CR58] Schneider J, Schneider P, Heyman J, Smart A (1999). Is transparency possible? The political-economic and epistemological implications of cold-war conspiracies and subterfuge in Italy. States and illegal practices: an overview.

[CR59] Sciarrone R, Storti L (2014). The territorial expansion of mafia-type organized crime. The case of the Italian mafia in Germany. Crime Law Soc Change.

[CR60] Sergi A (2014). Structure versus activity. Policing organized crime in Italy and in the UK. Distance and Convergence Policing: A Journal of Policy and Practice.

[CR61] Sergi A (2017). From mafia to organised crime a comparative analysis of policing models.

[CR62] Shortland A, Varese F (2016). State-building, informal governance and organised crime: the case of Somali piracy. Political Studies.

[CR63] Snyder R, Duran-Martinez A (2009). Does illegality breed violence? Drug trafficking and state-sponsored protection rackets. Crime Law Soc Change.

[CR64] Sobering K, Auyero J (2019). Collusion and cynicism at the urban margins. Latin America Research Review.

[CR65] The Guardian (2019) Venezuela’s Guaidó pictured with members of Colombian gang. Available at: https://www.theguardian.com/world/2019/sep/13/juan-guaido-faces-questions-over-links-toorganized-groups. Accessed 23/12/2019

[CR66] Tinti P, Reitano T (2017). Migrant, Refugee, Smuggler, Savoir.

[CR67] UNODC (2019) Colombia. Monitoreo de Territorios Afectados por Cultivos Ilícitos 2018. Bogotá

[CR68] Varese, F (2017) What is Organised Crime? In: Carnevale S, Forlati, S & Orsetta G (eds) Redefining Organised Crime. A Challenge for the European Union? (pp. 27–55). Oxford: Hart Publishing

[CR69] Villa R, Souza M (2019). Violent non-state actors and new forms of governance: exploring the Colombian and Venezuelan border zone. Journal of Human Security.

[CR70] Von Lampe K (2016). Organized crime. Analyzing illegal activities, criminal structures, and extra-legal governance.

[CR71] Zartman I (2010). Understanding life in the borderlands: boundaries in depth and motion.

